# Detection of COVID-19 Patients from CT Scan and Chest X-ray Data Using Modified *MobileNetV2* and *LIME*

**DOI:** 10.3390/healthcare9091099

**Published:** 2021-08-25

**Authors:** Md Manjurul Ahsan, Redwan Nazim, Zahed Siddique, Pedro Huebner

**Affiliations:** 1 Industrial and Systems Engineering, University of Oklahoma, Norman, OK 73019, USA; 2 Chemical, Biological & Materials Engineering, University of Oklahoma, Norman, OK 73019, USA; redwan.nazim-1@ou.edu; 3 School of Aerospace and Mechanical Engineering, University of Oklahoma, Norman, OK 73019, USA; zsiddique@ou.edu

**Keywords:** chest X-ray, CT scan, coronavirus, COVID-19, deep learning, imbalanced data, mixed-data, SARS-CoV-2, small data, explainable AI

## Abstract

The COVID-19 global pandemic caused by the widespread transmission of the novel coronavirus (SARS-CoV-2) has become one of modern history’s most challenging issues from a healthcare perspective. At its dawn, still without a vaccine, contagion containment strategies remained most effective in preventing the disease’s spread. Patient isolation has been primarily driven by the results of polymerase chain reaction (PCR) testing, but its initial reach was challenged by low availability and high cost, especially in developing countries. As a means of taking advantage of a preexisting infrastructure for respiratory disease diagnosis, researchers have proposed COVID-19 patient screening based on the results of Chest Computerized Tomography (CT) and Chest Radiographs (X-ray). When paired with artificial-intelligence- and deep-learning-based approaches for analysis, early studies have achieved a comparatively high accuracy in diagnosing the disease. Considering the opportunity to further explore these methods, we implement six different Deep Convolutional Neural Networks (Deep CNN) models—VGG16, MobileNetV2, InceptionResNetV2, ResNet50, ResNet101, and VGG19—and use a mixed dataset of CT and X-ray images to classify COVID-19 patients. Preliminary results showed that a modified MobileNetV2 model performs best with an accuracy of 95 ± 1.12% (AUC = 0.816). Notably, a high performance was also observed for the VGG16 model, outperforming several previously proposed models with an accuracy of 98.5 ± 1.19% on the X-ray dataset. Our findings are supported by recent works in the academic literature, which also uphold the higher performance of MobileNetV2 when X-ray, CT, and their mixed datasets are considered. Lastly, we further explain the process of feature extraction using Local Interpretable Model-Agnostic Explanations (LIME), which contributes to a better understanding of what features in CT/X-ray images characterize the onset of COVID-19.

## 1. Introduction

The novel coronavirus (SARS-CoV-2) global pandemic has represented one of humanity’s greatest challenges in modern history. For most of the now year-and-a-half long crisis, a vaccine, despite having accelerated development due to the global emergency, remained unavailable for most people. The advent of the new COVID-19 delta strain introduced another layer of concern as rates of transmission and resistance to select vaccines are notably high. According to recent guidelines from the US Center for Disease Control and Prevention (CDC), vaccinated individuals should continue to wear masks to prevent viral transmission and the infection of unvaccinated individuals [1]. Statistically, the number of affected individuals and casualties are astounding and alarming: 200,237,344 and 4,258,459, respectively, as of 3 August 2021 [2], with an associated mortality rate of about 2.13 percent. As a measure to reduce the spread of the virus—which transmits itself through close contact and respiratory droplets of infected individuals while talking, coughing, or sneezing—many countries prohibited any social gathering in community, work, and school, and forced citizens into mandatory lockdowns and quarantining. A key opportunity to minimize the spread is to correctly diagnose infected individuals; currently, real-time reverse transcription-polymerase chain reaction (RT-PCR) is used as a gold-standard test to diagnose the onset of COVID-19 [3,4]. However, the limitations surrounding the depth of our understanding regarding the nature of the virus, testing kits may be associated with a high error rate, approaching 30% [2]. Inaccurate testing has been credited as one of the many contributing factors of ineffective disease containment. As a result, researchers have proposed alternative approaches, such as chest X-ray- and CT-scan-based patient diagnosis as options to support the early identification of individuals potentially carrying the virus. Such techniques can take great advantage of current deep-learning- and artificial-intelligence (AI)-based methods applied to either small data [5,6,7,8] or large datasets [5,9,10]. For instance, Chen et al. (2020) proposed a UNet++ model using a small dataset containing 51 COVID-19 and 82 non-COVID-19 patients and achieved an accuracy of around 98.5% [6]. Similarly, Ardakani et al. (2020), used a small dataset of 108 COVID-19 and 86 non-COVID-19 patients to test ten different deep learning models and obtained a 99% accuracy overall [7]. Wang et al. (2020) proposed an inception-based model utilizing a comparatively large dataset, with 453 CT scan images being incorporated in the analysis, ultimately obtaining an accuracy of 73.1% [9]. However, along with lower accuracy, the model’s network activity and region of interest were not clearly explained. Lastly, Li et al. (2020) used a moderately large dataset containing 4356 chest CT images of pneumonia patients, of which 1296 were confirmed COVID-19 cases, and obtained 96% accuracy with the proposed COVNet model [5].

**Figure 1:**
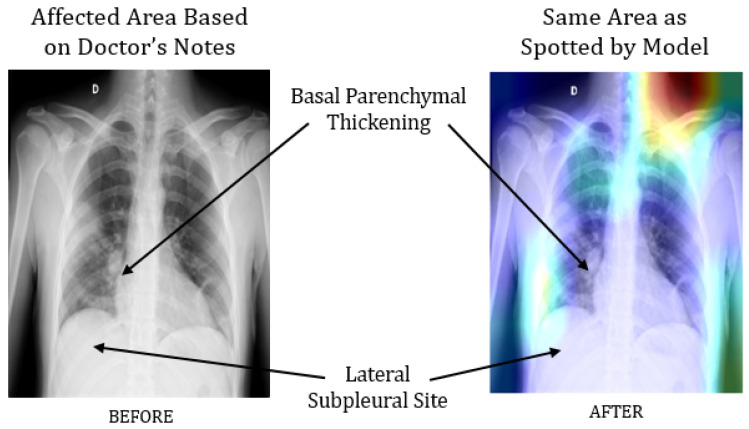
Comparison between a chest X-ray image analyzed by a doctor and a modified VGG16 model, wherein its layer “Block_4” drew particular attention to the collarbone and upper shoulder [14].

In parallel, several studies explored and recommended screening COVID-19 patients using chest X-ray images instead—notable contributions can be found in [11,12,13]. For instance, Hemdan et al. (2020) worked on a small dataset, comprising only 50 images, and demonstrated an accuracy of 90% and 95% in predicting COVID-19 patients from chest X-ray images using VGG19 and ResNet50 models, respectively [11]. Using a dataset of 100 images, Narin et al. (2020) distinguished COVID-19 patients from those with pneumonia with 86% accuracy [13]. However, due to the relatively small dataset, questions remain regarding the model’s stability and interpretability. To address these issues, our previous work has focused on representing the performance of different deep learning models with 95% confidence intervals, so as to understand and better interpret their performance on small datasets. For example, with a data pool of 50 chest X-ray images, we found that InceptionResNetV2 models identify COVID-19 patients with 97% accuracy, but with the Wilson score method representing an accuracy in the range of 68.1% to 99.8%. Besides, the study also revealed that deep CNN-based architecture, such as VGG16 and ResNet50, often extract unnecessary features from the images, especially when applied on very small datasets. For instance, a modified VGG16 model identified 97% of COVID-19 patients correctly, but the model architecture emphasized a significant amount of features in the region of the collarbone and upper shoulder instead of the region of interest on the chest and lungs, as shown in Figure 1.

**Figure 2:**
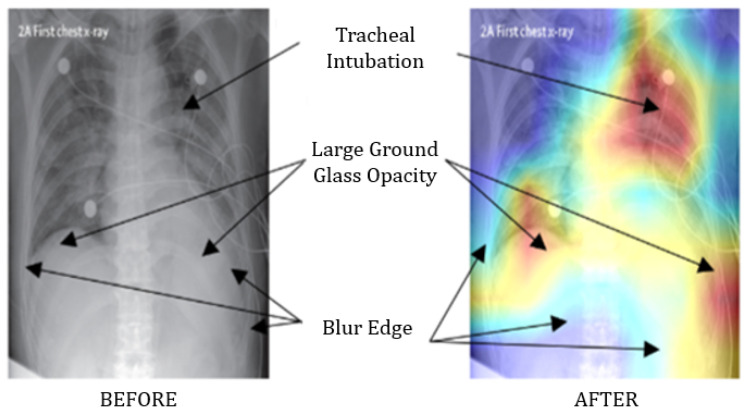
The extraction of unnecessary or irrelevant features was reduced significantly following the analysis of a larger dataset [14].

However, a significant improvement was observed utilizing a comparatively larger dataset of 1845 chest X-ray images, which ultimately demonstrated higher accuracy [14]. Models trained with such big data convey the advantages over small data by reducing unnecessary or irrelevant feature detection on chest X-ray images, as shown in Figure 2.

Researchers often train their models with large chest X-ray image datasets [15,16] in order to develop a robust model. For example, 6505 images with a data ratio of 1:1.17 were utilized by Brunese et al. (2020), wherein 3003 images were patients with COVID-19 symptoms, and 3520 were labeled as “other patients” for the purposes of that study [15]. Ghoshal and Tucker (2020) used a dataset of 5941 images and achieved 92.9% accuracy [16]. However, neither study assessed or discussed how their proposed models would perform with highly imbalanced data containing unequal class ratio. On that note, Apostol, Oztuk, and Khan (2020) considered an imbalanced dataset of 284 COVID-19 and 967 non-COVID-19 patient chest X-ray images and achieved 89.6% accuracy using a CNN-based Xception model [17]. Despite the demonstrated potential, challenges associated with the unequal data ratio, such as the risk of overfitting or underfitting during the training stages, were not explored in detail. Considering those opportunities and the rapid spread of a transmittable disease such as COVID-19, we recognize that existing resources and methodologies are not alone sufficient to serve as a reliable means of diagnosis during the early stages of a rapidly spreading pandemic. Thus, instead of using only chest CT or X-ray-based screening, a better solution lies in integrating the usage of both techniques. A few advantages of this proposed method include more patients being able to get tested, and less reliability on COVID 19 testing kits. We explore this opportunity and investigate a reliable and explainable AI-based COVID-19 screening system that can identify symptomatic patients from widely available medical image data. In this study, we apply and evaluate the performance of several AI-based models with a mixed dataset containing both chest CT and X-ray images. We summarize our main contributions as follows:

- Implementation and evaluation of six different deep CNN models (VGG16 [18], InceptionResNetV2 [19], ResNet50 [20], MobileNetV2 [21], ResNet101 [22], and VGG19 [18]) to detect COVID-19 patients using a mixed dataset of chest CT and X-ray images;
- A detailed analysis of the results obtained and comparison with the performance of the same models being applied to independent datasets of either CT scans or X-ray images;
- Finally, we explain the models’ predictability considering top features with Local Interpretable Model-Agnostic Explanations (LIME).

## 2. Research Methodology

**Figure 3:**
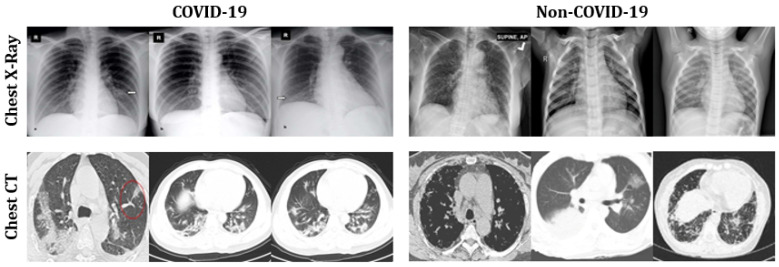
Representative sample images of chest X-rays and CT scans used in the mixed dataset adopted for analysis.

Table 1 summarizes our adopted dataset [23], which contains both CT scans (200 COVID-19 and 200 Non-COVID-19) and chest X-rays (1583 COVID-19 and 608 Non-COVID-19) of patients expressing pneumonia symptoms. We dedicated 80% of the data for training and the remaining 20% for testing. Figure 3 presents a set of representative images used in the analysis.

### 2.1. Using Pre-Trained Convolutional Networks

We used six different pre-trained ConvNets: VGG16, MobileNetV2, ResNet50, ResNet101, InceptionResNetV2, and VGG19. A comprehensive explanation of the network’s architecture can be found in [24]. Each model is developed with the advantages of transfer learning. The modified architecture was developed using the following steps:

1. Models are initiated with the pre-trained network without a fully connected (FC) layer;
2. A new layer is added, containing “Maxpool” and “softmax” as activation functions and appended with the network’s primary architecture;
3. The convolutional weights are kept frozen and only the new FC layers are trained with the new CNN architecture.

A similar procedure was applied for the other five proposed deep CNN models. The constructed CNN architecture has the following sequence: AveragePooling2D (Poolsize= (4.4))—Flatten—Dense—Dropout (0.5)—Dense (Activation = “Softmax”). Three parameters, specifically batch size, epochs, and learning rate (as suggested by [25,26]), are considered for model optimization. We adopted the commonly employed grid search method [27] to fine tune parameters. At first, the following were chosen at random:

Batch size = [20, 30, 40, 50, 60];

Number of epochs = [20, 25, 30, 35, 40];

Learning rate = [0.001, 0.01, 0.1].

Following the final computation, best results were obtained with the following:

Batch size = 50;

Number of epochs = 35;

Learning rate = 0.001.

Adaptive learning rate optimization, also known as Adam [28,29], was used as an optimization algorithm as used in previous works [14]. The experimental procedure was run twice, and the results were obtained by averaging the two results. The statistical analysis was evaluated in terms of accuracy, precision, recall, and f-1 score [30], as defined below:

(1) $$Accuracy=\frac{t_{p}+t_{n}}{t_{p}+t_{n}+f_{p}+f_{n}}$$

(2) $$Precision=\frac{t_{p}}{t_{p}+f_{p}}$$

(3) $$Recall=\frac{t_{p}}{t_{n}+f_{p}}$$

(4) $$F1=2\times \frac{Precision\times Recall}{Precision+Recall}$$

where,

True positive ($t_{p}$) = COVID-19 infectious patients classified as patients;

False Positive ($f_{p}$) = Healthy people classified as COVID-19 patients;

True Negative ($t_{n}$) = Healthy people classified as healthy;

False Negative ($f_{n}$) = COVID-19 infectious patients classified as healthy.

### 2.2. LIME as Explainable AI

The overall prediction was interpreted using LIME, a procedure that allows the understanding of the input features of the deep learning models which affect its predictions. LIME is regarded as one of the few methodologies that works well with tabular data, text, and images, and is extensively employed for its reliability in explaining the intricacies of image classification [31]. For image classification, LIME creates superpixels. Superpixels are the result of image over-segmentation. Superpixels store more data than pixels and are more aligned with image edges than rectangular image patches [32]) for the primary prediction. Table 2 shows the parameters used to calculate the superpixel during this experiment.

## 3. Results

Table 3 presents a summary of the performance of all models on the training and test sets along with a 95% confidence interval. MobileNetV2 outperformed all models in terms of accuracy, precision, recall, and f-1 score. Contrarily, the ResNet50 model showed the worst performance considering all measures.

**Figure 4:**
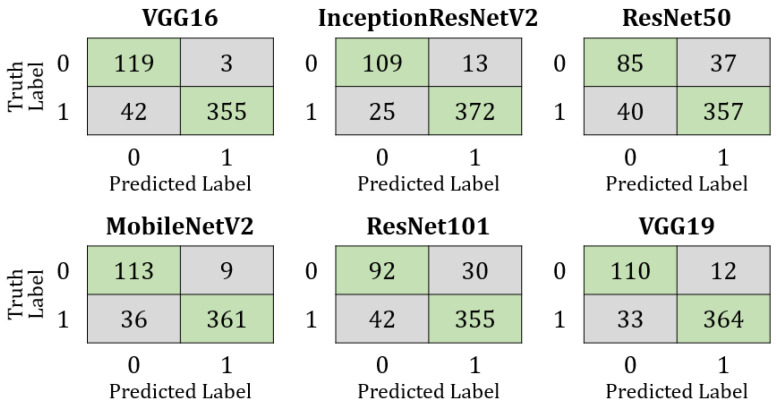
Confusion matrices of all models applied to the mixed test dataset.

To better understand the overall performance of each model during the prediction stage on the test set, Figure 4 presents a set of confusion matrices. The test set contained a combination of 519 chest X-ray and CT scan images (122 COVID-19 and 397 Non-COVID-19). It can be detected that MobileNetV2 and VGG19 correctly classified the maximum number of COVID-19 and non-COVID-19 patients, whereas ResNet50 expressed the worst performance with the maximum number of misclassified samples compared to any other model.

**Figure 5:**
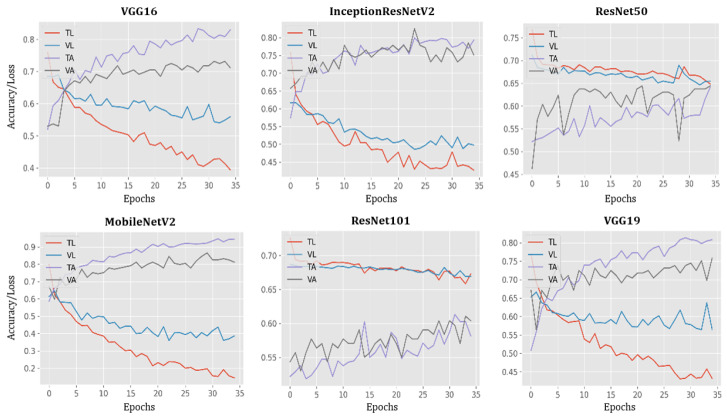
Plots of model accuracy and loss following each epoch applied to both training and testing datasets; TL = training loss; VL = validation loss; TA = training accuracy; VA = validation accuracy.

The performance of all models during training and testing, per each epoch, are presented in Figure 5. In this case, the accuracy of VGG16, MobileNetV2, and VGG19 models reached 100% while loss decreased by nearly 100% at epoch 35.

### AUC-ROC Curve

**Figure 6:**
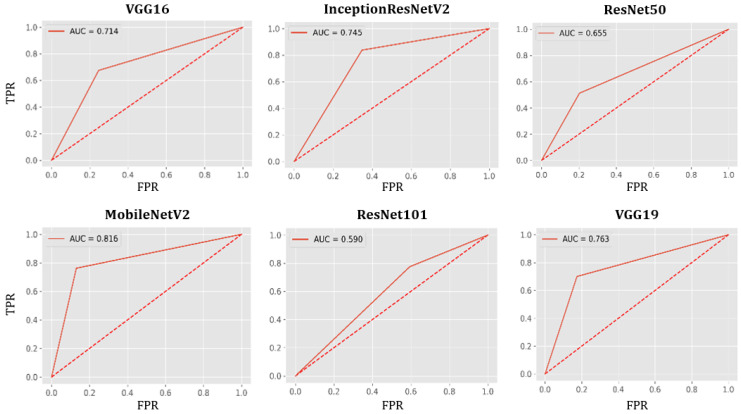
AUC-ROC curves for all models using the test set.

In Figure 6, measures of the Area Under the Curve (AUC) of the Receiver Characteristic Operator (ROC) are plotted for each model with the true positive rate (TPR) in the vertical axis and false positive rate (FPR) in the horizontal axis, applied to the test set. MobileNetV2 shows the best performance (AUC=0.816), while ResNet101 shows the worst (AUC=0.590).

**Figure 7:**
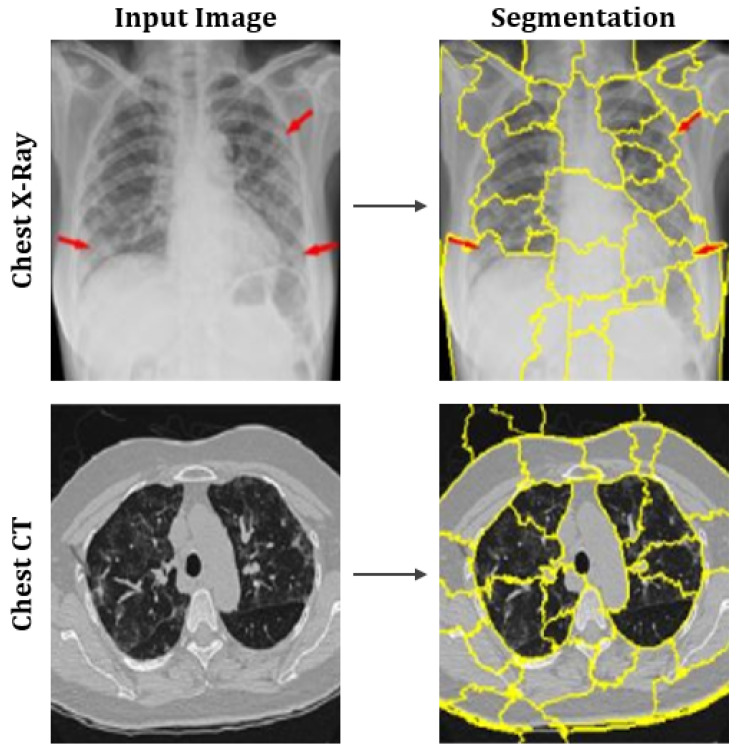
Representation of superpixels on sample images of chest X-rays and CT scans.

Figure 7 shows the output after computing the superpixels on sample CT scan and chest X-ray images.

**Figure 8:**
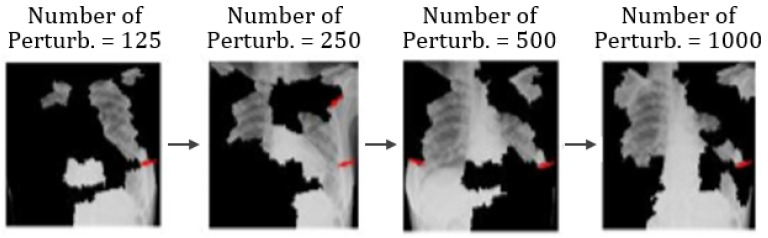
Example of the varying number of features as the number of perturbation changes.

Additionally, Figure 8 shows different image conditions in terms of perturbation vectors and perturbation images. Figure 8 illustrates that the number of features varies with the number of perturbations.

**Figure 9:**
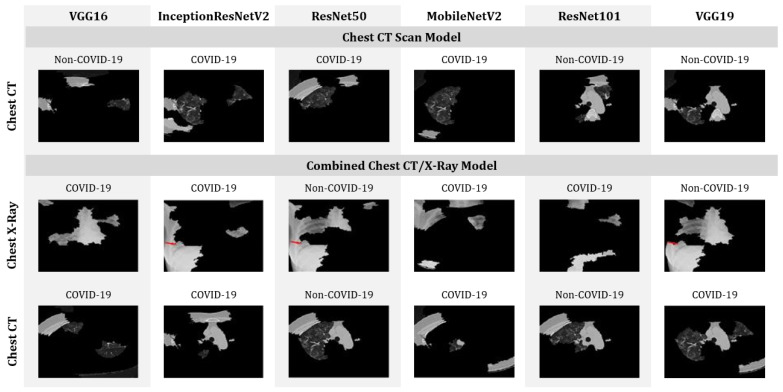
Top four features that enabled the identification of COVID-19 patients from CT-scan-only and mixed datasets.

The distance metric or cosine metric with a kernel width of 0.25 is used to understand the distance difference between each perturbation and the original image. A linear model is used for the proposed model’s explanations. Additionally, the coefficient was found for every superpixel in the picture which represents the strength of a superpixel’s impact on predicting COVID-19 patients. Finally, top features (only four features are considered for the purposes of this study) are sorted to determine the most essential superpixel, as shown in Figure 9. The features and the prediction were addressed together during this study. As shown in Figure 9, models, such as VGG16, MobileNetV2 and VGG19 trained with CT scan images incorrectly classified COVID-19 patients as Non-COVID-19 patients. On the other hand, while analyzing combined models, ResNet50 shows the worst performance by misclassifying both CT and chest X-ray images.

## 4. Discussion

In this study, six different deep-learning-based models were proposed and evaluated for their ability to distinguish between patients with and without COVID-19, with demonstrated advantages of tests conducted on combined datasets, comprising both CT scan and X-ray images (as opposed to a singular point of reference with only CT scans or X-rays). Among all proposed models, MobileNetV2 achieved an accuracy of 95 ± 1.12% depending on the dataset applied. A summary of the accuracy of all six models, considering the CT scan, chest X-ray, and the mixed dataset is presented in Table 4. Other than MobileNetV2, the VGG16 model demonstrates higher performance on X-ray dataset by achieving an accuracy of 98.5% ± 1.19%, which outperforms many studies in the current literature. For example, Wang and Wong (2020) [9] and Khan et al. (2020) [33] used CNN-based approaches to detect the onset of the COVID-19 disease using chest X-ray images and achieved an accuracy of 83.5% and 89.6%, respectively. In comparison, as previously stated, our proposed VGG16 and MobileNetV2 models achieved an accuracy of around 98.5% ± 1.19%.

In Table 5, the accuracy of different deep learning models used in previous studies are compared (where CT scan images were used for the experiment) with the models of this study in consideration of different database sizes. Here, an accuracy of 98.5% ± 1.19% was achieved using 400 images with the MobileNetV2 model. These results outperform the referenced literature [34,35], which used large datasets containing 4356 and 1065 images, respectively. In contrast, Butt et al. (2020) used a CNN-based approach, specifically a ResNet23 model to detect the onset of COVID-19 disease using chest CT scan images and achieved an accuracy of around 86.7% [10]. Jin et al. (2020) used 1882 CT scan images and achieved an accuracy of 94.1% [36].

It is relevant to emphasize that none of the referenced literature considered a mixed-dataset, which hinders a direct comparison with the results of this study. However, preliminary computational results on a mixed dataset indicated that a modified MobileNetV2 model is capable of differentiating between patients with COVID-19 symptoms with an accuracy of 95% ± 1.12%. Additionally, analyzing the proposed models with LIME illustrated MobileNetV2’s contribution to successfully characterizing the onset of COVID-19 by recognizing essential features in CT/X-ray images.

The primary goal of this study was to develop an integrated system that can detect patients with COVID-19 symptoms from a dataset containing CT scan, chest X-ray, or a combination of CT scan and chest X-ray images of potential COVID-19 patients. At this stage, the scope of the current literature in this field of work remains narrow and often does not consider combined CT scan and chest X-ray image datasets with explainable AI. Here, predicted features were identified with LIME to understand the models’ decision-making process. Going forward, results of studies such as the one herein presented must be verified in consultation with healthcare experts. In addition, future work can take advantage of evaluating how other interpretable models could be used with mixed datasets in an attempt to validate the overall predictions presented here.

## 5. Conclusions

In this study, we evaluated six different deep learning models on a mixed dataset of CT scan and chest X-ray images for their ability to identify COVID-19 patients. We revealed that a modified MobileNetV2 can achieve an accuracy of 95% on that task. We have also used Local Interpretable Model-Agnostic Explanations (LIME) to interpret and validate our predictions. The findings of the proposed models should provide some insights to researchers and practitioners regarding the application of explainable AI on screening COVID-19 patients based on chest X-ray and CT-scan images. Next steps which would build on the efforts of our work include developing user-friendly mobile apps/web-based COVID-19 screening systems using MobileNetV2 models and creating decision support systems along with numerical (i.e., age, gender) and categorical (findings, health conditions) data. Opportunities also lie in utilizing other image processing techniques, such as fuzzy entropy and divergence, so as to more precisely recognize edges and contours of X-rays and CT images [39,40]. 

## Figures and Tables

**Table 1:** Summary of the mixed dataset used in the analysis, including training and test sets.

Dataset	Label	Train			Test		
		Chest X-ray	CT scan	Total	Chest X-ray	CT scan	Total
Mixed Data	COVID-19	486	160	646	122	40	162
	Non-COVID-19	1266	160	1426	317	40	357
Total		1752	320	2072	439	80	519

**Table 2:** Superpixel calculation parameters.

Function	Value
Kernel size	200
Maximum distance	200
Ratio	0.2

**Table 3:** COVID-19 screening performance of all models using a mixed dataset, presented with 95% confidence intervals (CI, α=0.05). $T_{a}$—Training Set; $T_{s}$—Test Set.

Algorithm	Accuracy (%)			Precision (%)			Recall (%)			F-1 Score (%)		
	T${}_{a}$	T${}_{s}$	CI	T${}_{a}$	T${}_{s}$	CI	T${}_{a}$	T${}_{s}$	CI	T${}_{a}$	T${}_{s}$	CI
VGG16	95	91	93 ± 1.4	95	93	94 ± 1.3	95	91	93 ± 1.4	95	92	93.5 ± 1.34
InceptionResNetV2	94	93	93.5 ± 1.34	95	93	94 ± 1.3	94	93	93.5 ± 1.34	94	93	93.5 ± 1.35
ResNet50	88	85	86.5 ± 1.86	87	85	86 ± 1.89	88	85	86.5 ± 1.86	87	85	86 ± 1.89
MobileNetV2	99	91	95 ± 1.2	99	92	95.5 ± 1.13	99	91	95 ± 1.2	99	91	95 ± 1.2
ResNet101	88	86	87 ± 1.83	88	87	87.5 ± 1.80	88	86	87 ± 1.83	88	86	87 ± 1.83
VGG19	94	91	92.5 ± 1.43	94	92	93 ± 1.4	94	91	92.5 ± 1.43	94	92	93 ± 1.4

**Table 4:** Top-performing models in terms of accuracy and different datasets adopted.

Dataset	Datasize	Model	Accuracy (%)
X-ray	400	VGG16	98.5 ± 1.191
		MobileNetV2	98.5 ± 1.191
CT-Scan	400	MobileNetV2	94 ± 2.327
Mixed-data	2591	MobileNetV2	95 ± 1.12

**Table 5:** Comparison between previous studies found in the literature and our present study.

Reference	Model	Dataset Size	Accuracy
Li et al. (2020) [34]	ResNet50	4356	90%
Wang et al. (2021) [35]	Inception-M	1065	74%
Zhang et al. (2020) [37]	ResNet50	1531	90%
Song et al. (2020) [38]	ResNet50	274	86%
Chen et al. (2020) [6]	UNet${}^{++}$	133	98.5%
Jin et al. (2020) [36]	CNN	1882	94.1%
This study	MobileNetV2	400	98.5% ± 1.19%

## Data Availability

Not applicable.
